# Efficacy of direct current generated by multiple-electrode arrays on F3II mammary carcinoma: experiment and mathematical modeling

**DOI:** 10.1186/s12967-020-02352-6

**Published:** 2020-05-07

**Authors:** Narciso Antonio Villar Goris, Jorge Luis García Rodríguez, Maraelys Morales González, Beatriz Olivares Borges, Dasha Fuentes Morales, Enaide Maine Calzado, Antonio Rafael Selva Castañeda, Leonardo Mesa Torres, Juan Ignacio Montijano, Victoriano Gustavo Sierra González, Daniel Jay Pérez, Oscar Ortiz Posada, Janet Avellanet Martínez, Arlem García Delgado, Karina García Martínez, Mayrel Labrada Mon, Kalet León Monzón, Héctor Manuel Camué Ciria, Luis Enrique Bergues Cabrales

**Affiliations:** 1 Universidad Autónoma de Santo Domingo, Santo Domingo, República Dominicana; 2 Universidad Católica del Cibao, La Vega, República Dominicana; 3 Departamento de Investigación e Innovación, Centro Nacional de Electromagnetismo Aplicado, Dirección de Ciencia e Innovación , Universidad de Oriente, Ave. Las Américas s/n, Santiago de Cuba, 90400 Cuba; 4 Departamento de Farmacia, Facultad de Ciencias Naturales y Exactas, Universidad de Oriente, Santiago de Cuba, Cuba; 5 Laboratorio Farmacéutico Oriente, Santiago de Cuba, Cuba; 6 Centro Nacional para la Producción de Animales de Laboratorio, La Habana, Cuba; 7 Departamento de Telecomunicaciones, Facultad de Ingeniería Eléctrica, Universidad de Oriente, Santiago de Cuba, Cuba; 8 Instituto Universitario de Investigación de Matemáticas y Aplicaciones, Universidad de Zaragoza, Saragossa, Spain; 9 Grupo de las Industrias Biotecnológica y Farmacéuticas (BioCubaFarma), La Habana, Cuba; 10 Centro de Inmunología Molecular, La Habana, Cuba

**Keywords:** Modified Gompertz equation, Tumor growth kinetics, Highly aggressive and metastatic primary F3II mammary carcinoma, Electrochemical therapy, Array of multiple-electrodes

## Abstract

**Background:**

The modified Gompertz equation has been proposed to fit experimental data for direct current treated tumors when multiple-straight needle electrodes are individually inserted into the base perpendicular to the tumor long axis. The aim of this work is to evaluate the efficacy of direct current generated by multiple-electrode arrays on F3II mammary carcinoma that grow in the male and female BALB/c/Cenp mice, when multiple-straight needle electrodes and multiple-pairs of electrodes are inserted in the tumor.

**Methods:**

A longitudinal and retrospective preclinical study was carried out. Male and female BALB/c/Cenp mice, the modified Gompertz equation, intensities (2, 6 and 10 mA) and exposure times (10 and 20 min) of direct current, and three geometries of multiple-electrodes (one formed by collinear electrodes and two by pair-electrodes) were used. Tumor volume and mice weight were measured. In addition, the mean tumor doubling time, tumor regression percentage, tumor growth delay, direct current overall effectiveness and mice survival were calculated.

**Results:**

The greatest growth retardation, mean doubling time, regression percentage and growth delay of the primary F3II mammary carcinoma in male and female mice were observed when the geometry of multiple-pairs of electrodes was arranged in the tumor at 45, 135, 225 and 325^o^ and the longest exposure time. In addition, highest direct current overall effectiveness (above 66%) was observed for this EChT scheme.

**Conclusions:**

It is concluded that electrochemical therapy may be potentially addressed to highly aggressive and metastic primary F3II murine mammary carcinoma and the modified Gompertz equation may be used to fit data of this direct current treated carcinoma. Additionally, electrochemical therapy effectiveness depends on the exposure time, geometry of multiple-electrodes and ratio between the direct current intensity applied and the polarization current induced in the tumor.

## Highlights

- Multiple-pairs of electrodes are experimentally verified for the first time in the literature.
- Different geometries of multiple-electrodes and long exposure time can be addressed for electrochemical therapy.
- Modified Gompertz equation can be applied to any geometry of multiple-electrodes.
- Exposure time and electrode array geometries are included in the parameters of modified Gompertz equation.

## Background

Antitumor effectiveness of the electrochemical therapy (EChT) with low-level direct current (DC) is demonstrated in in vitro [1], preclinical [2] and clinical [3] studies. Nevertheless, it is poorly understood how tumor growth kinetics (TGK) is affected by DC application.

The modified Gompertz equation (MGE) to simulate and fit the different responses of experimental tumors after DC application is suggested by Cabrales et al. [4], such as: disease progression (DP), stable disease (SD), partial response (PR) and complete response (CR). The data of Ehrlich and fibrosarcoma Sa-37 primary tumors [3] and highly aggressive and metastatic primary F3II mammary carcinoma [5] are adequately fitted with this equation. This validation of MGE is carried out for different values of DC intensity (i) and time of exposure of it (t_exp_), fixing the same electrode array geometry. Additionally, the stationary partial response [4], other findings [6] and how space–time distribution of the tumor density changes for each tumor response post-treatment [7] is revealed from the simulation of MGE. These theoretical and experimental results are valid when two or more collinear electrodes with alternating polarities inserted perpendicular to the larger diameter of the tumor are used.

In EChT, an important and interesting issue is to search electrode array geometries that maximizes the tumor volume with minimum damage to the organism. For this, multiple-straight needle electrodes inserted individually in the tumor (MSNEII) are recommended for EChT: MSNEII inserted colineally along the major axis of the tumor (MSNEII_c_) [2, 3, 5, 6] or MSNEII inserted non-colineally anywhere of the tumor (MSNEII_nc_) [8]. MSNEII_c_ is the most used in preclinical [5] and clinical studies [3]; nevertheless, they have not given a definitive solution to the cancer cure. Therefore, efforts are addressed to propose new MSNEII_nc_.

The use of multiple-pairs of electrodes (MPE) for EChT treated tumors is suggested by Calzado et al. [9]. High tumor damage percentages with the minimum damage to the organism are theoretically revealed for simulations of MSNEII_nc_ [8] and MPE [9], being noticeable for MPE. Nevertheless, the antitumor effectiveness and effects in the organism generated by these non-colineal electrode arrays are not reported in the literature. Additionally, we are not aware of the use of MGE to fit TGK treated with these multiple-electrode arrays. Therefore, the aim of this work is to evaluate the efficacy of direct current generated by multiple-electrode arrays on F3II mammary carcinoma that grow in the male and female BALB/c/Cenp mice, when collinear MSNEII_c_ and MPE are inserted in the tumor.

## Methods

### Experiment

A longitudinal and retrospective preclinical study was carried out between September–November 2017. The sarcomatoid mammary carcinoma cell line F3II that grow in BALB/c/Cenp mice was provided by the Centro de Inmunología Molecular (La Habana, Cuba). The characteristics of F3II mammary carcinoma were reported in [5, 10].

Stock F3II cells were conserved in minimal essential medium (MEM 41500, Gibco-BRL, Grand Island, NY) supplemented with 10% fetal bovine serum (Merck KGaA, Darmstadt, Germany), 2 mM glutamine (Merck KGaA, Darmstadt, Germany), 80 mg/ml Gentamycin (Merck KGaA, Darmstadt, Germany), and 20 mg/ml tetracycline (Merck KGaA, Darmstadt, Germany) in monolayer culture. For harvesting, cells were trypsinized using standard procedures. The cell viability was quantified by Trypan blue dye exclusion test (Merck KGaA, Darmstadt, Germany) and over 95%. Cell count was carried out under a microscope (model CX31, Olympus, Tokyo, Japan).

One hundred and sixty BALB/c/Cenp male and female mice (80 males and 80 females), 6–7 week old and 18–20 g weight were initially used. These mice were supplied by the Centro Nacional para la Producción de Animales de Laboratorio (CENPALAB, La Habana, Cuba). The experiment was conducted under a protocol approved by the Institutional Animal Care and Use Committee of CENPALAB (Registration number 16/17, code AETM0917, 17 May 2017), guidelines Animal Ethic Comission of República de Cuba and Council Directive 86/609/ECC of 24 November 1986, which followed guidelines for the welfare of animals in experimental neoplasia [11]. Each mouse was identified by dyeing with picric acid. Following the inoculation of tumor cells in the BALB/c/Cenp mice, daily meticulous clinical observations (including cage-side and handheld observations) were conducted throughout the study on all mice to monitor their general health states. Additionally, these observations were made to determine if significant clinical abnormalities or death were present in mice from any of the experimental groups.

2x10^5^ F3II sarcomatoid mammary carcinoma cells in 0.2 ml of 0.9% NaCl (sodium chloride) in the right dorsolateral region were inoculated in all mice. NaCl was supplied by the Laboratorios Biológicos Farmacéuticos (LABIOFAM, La Habana, Cuba). The latency time of local F3II tumor was monitored by palpation, which was done three times per week. The major and minor tumor diameters, in millimeters (mm), were measured with a venier caliper (Model 530-104, Mitutoyo, Tokyo, Japan) twice a week in order to record the evolution of each syngenic F3II tumor without sacrificing any animal. The volume of each individual tumor was computed by means of the formula V = πab^2^/6, in which a was the major diameter of this tumor histological variety and b its minor diameter.

Mice were housed in clear standard polycarbonate cages (1264C, TECNIPLAST, Varese, Italy) of 206 mm^2^ × 12 cm (5 animals/cage) with autoclaved hard wood-shavings as bedding. They were maintained under automatically controlled environmental conditions (NODOREM^®^, Instituto Cubano de Investigaciones Digitales, La Habana, Cuba): temperature of 22 ± 2 °C, 60–80% relative humidity, 12-h light/dark (light 7:00–19:00) and a room air exchange of 12–18 times/h. Animals were provided Certified Rodent Diet EMO1004 (ALYCO^®^, CENPALAB, La Habana, Cuba) in granulated form. Feed and water were sterilized by autoclaving (HS66, GETINGE, Gothenburg, Sweden) and available ad libitum. Autoclaving was made at 120 °C for 60 min for water and at 120 °C for 20 min for feed and bedding. Animals bedding were daily changed.

Body weight of each mouse was determined with a precision balance (Cubis^®^ MSU, Sartorius, Goettingen, Germany) prior to inoculation of cells and then weekly. Furthermore, survival checks for morbidity and mortality were made twice per day. Any animal found dead or moribund was subjected to gross necropsy.

**Fig. 1:**
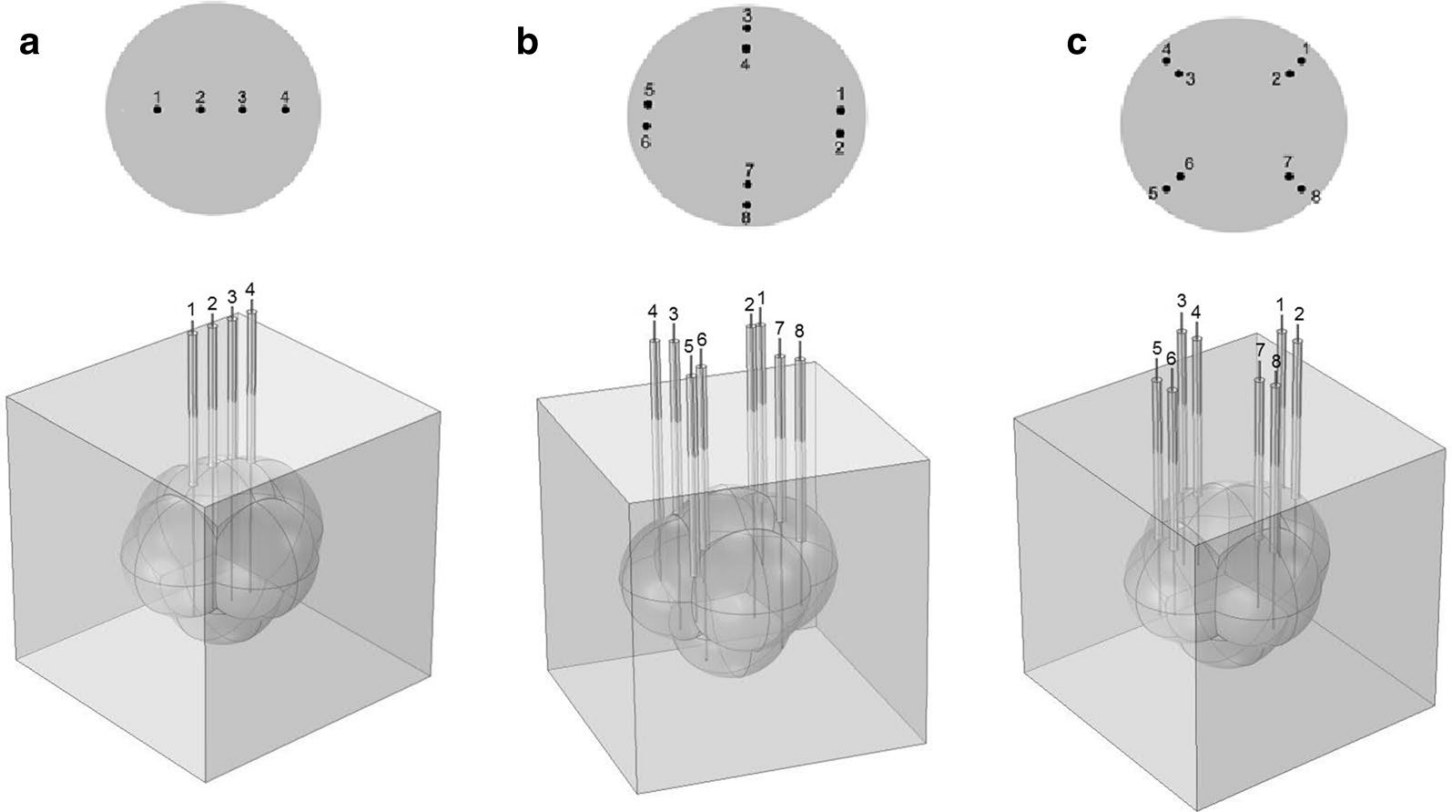
Schematic representation of different multiple-electrodes arrays inserted in a solid tumor. **a** Four collinear electrodes with alternating polarities inserted perpendicularly along the largest diameter of the tumor. Electrodes 1 and 3 (positive) and 2 and 4 (negative) were identified (C-I). **b** Four electrode pairs were arranged in the tumor to 45° (electrode pair 1–2), 135° (electrode pair 3–4), 225° (electrode pair 5–6) and 325° (electrode pair 7–8). Electrodes 1, 4, 5 and 8 were positives whereas electrodes 2, 3, 6 and 7 negatives (C-II). **c** Four electrode pairs were arranged in the tumor to 0° (electrode pair 1–2), 90° (electrode pair 3–4), 180° (electrode pair 5–6) and 270° (electrode pair 7–8). Electrodes 2, 3, 5 and 8 were positives whereas electrodes 1, 4, 6 and 7 negatives (C-III). Furthermore, the direction of insertion depth along the z-direction and the central plane represented by z = 0 were shown for each geometry of electrode array

One configuration of MSNEII_c_ and two of MPE were chosen from the simulations reported by Calzado et al. [9]. MSNEII_c_ was formed by four collinear electrodes with alternating polarities inserted perpendicularly along the largest diameter of the tumor, named C-I. For C-I, electrodes 1 and 3 were positives whereas electrodes 2 and 4 negatives (Fig. 1a).

The two configurations of MPE were formed by four electrode pairs inserted into the tumor. The first configuration of MPE, named C-II, was formed by electrode pairs arranged to 45° (electrode pair 1–2), 135° (electrode pair 3–4), 225° (electrode pair 5–6) and 325° (electrode pair 7–8), as shown in Fig. 1b. For C-II, electrodes 1, 4, 5 and 8 were positives whereas electrodes 2, 3, 6 and 7 negatives. The second configuration of MPE, named C-III, was formed by electrode pairs arranged to 0° (electrode pair 1–2), 90° (electrode pair 3–4), 180° (electrode pair 5–6) and 270° (electrode pair 7–8), as shown in Fig. 1c. For C-III, electrodes 2, 3, 5 and 8 were positives whereas electrodes 1, 4, 6 and 7 negatives.

For each pair of electrodes in each configuration of MPE, its angle was measured with respect to the largest tumor diameter counterclockwise. It was theoretically reported that the highest percentage of tumor damage was induced by C-II [9]. Therefore, C-II was the most used in this study.

The distance between electrodes was fixed at 0.5 cm for C-I, C-II and C-III. The AISI 316L austenitic stainless steel was chosed as electrode material for DC treatment (see details in [5]). The diameter of each electrode was 0.7 mm and its length 68.5 mm.

After all electrodes were inserted, they were connected to the DC source. The ONCOCED^®^ B&E-01 device was used for EChT, which was applied when the initial volume of the tumors (V_o_) was approximately 0.5 cm^3^ (zero day). This tumor size was reached 31 days after their cells were inoculated in mice. DC intensity and voltage were continuously monitored during EChT application.

Once tumor cells were inoculated into mice, animals were randomly divided into eight experimental groups: a first control group, in which C-I was used but DC was not applied (CG1); a second control group, in which C-II was used but DC was not applied (CG2); a first group treated with 2 mA for 10 min and C-I (TG1); a second group treated with 6 mA for 20 min and C-II (TG2); a third group treated with 2 mA for 10 min and C-III (TG3); a fourth group treated with 2 mA for 10 min and C-II (TG4); a fifth group treated with 6 mA for 10 min and C-II (TG5) and a sixth group treated with 10 mA for 10 min and C-II (TG6). These groups for females were defined CG1-F, CG2-F, TG1-F, TG2-F, TG3-F, TG4-F, TG5-F and TG6-F whereas CG1-M, CG2-M, TG1-M, TG2-M, TG3-M, TG4-M, TG5-M and TG6-M for the males.

Each experimental group was initially formed by 10 females and 10 males. The reduction (one of the criteria of the 3R [12, 13]) was taken into account to establish the number of animals, by experimental group.

Anesthesia before inserting the electrodes into the tumor was administered to mice. Anesthesia consisted of a mixture of 2.5 ml of Ketamine (Liorad, La Habana, Cuba), 1 ml of Atropine (LABIOFAM, La Habana, Cuba) and 2 ml of Diazepam (Empresa Laboratorios AICA del Grupo de las Industrias Biotecnólogica y Farmacéutica, La Habana, Cuba). This mixture was administered intraperitoneally (0.1 ml per 20 g of weight). The injection area was disinfected with 70% ethanol (Ministerio de Salud Pública, La Habana, Cuba), before and after administering anesthesia. All animals were placed in an isothermal blanket at 37 °C for recovery from anesthesia.

Control and treated groups were maintained under the same experimental conditions. Electrodes were inserted in all mice; nevertheless, the mice in the control groups did not receive DC. All mice were located on an isothermal blanket at 37 °C for recovery from anesthesia. Daily evaluations of the clinical signs and symptoms, morbidity and mortality of each animal were made until the moment of euthanasia. Mice were bled by the femoral vein, prior to anesthesia with diethyl ether (Merck, Darmstadt, Germany). After, these animals were sacrificed by cervical dislocation. Euthanasia of these mice was governed by the Standard Operating Procedure established in the CENPALAB (Edition 2003–2018).

Three kinetc parameters for each individual F3II mammary carcinoma were calculated, such as: regression percent (in %), mean doubling time (in days) and growth delay (in days). The first parameter was calculated by $\left[ {{{\left( {V_{\hbox{min} } - V_{o} } \right)} \mathord{\left/ {\vphantom {{\left( {V_{\hbox{min} } - V_{o} } \right)} {V_{o} }}} \right. \kern-0pt} {V_{o} }}} \right]\,100\,\%$, where V_min_ was interpreted as the minimum tumor volume reached after DC application. The second parameter was defined as the time required for that this tumor type reaches a twofold increase of its initial volume. The third parameter was calculated by means of the ratio between mean doubling time of treated group and mean doubling time of its corresponding control group.

The individual tumor response after DC treatment (DP, SD, PR o CR) was documented in this study. Additionally, overall EChT effectiveness (PR + CR) was reported, as suggested González et al. [5].

### Modified Gompertz equation

The analysis of the entire TGK in the same mice and in two parts was suggested by González et al. [5]: the first part comprised from the moment of inoculation of the tumor cells in the host until the day that DC was applied, called TGK_1_. The second part included from the moment of DC application until the day of sacrifice of the mice for ethical reasons [11], called TGK_2_. This individual analysis of the TGK was done for each male and female. For this, MGE (Eq. 1) was used to fit experimental data for individual F3II mammary carcinoma treated with C-I, C-II and C-III and given by

1 $$V^{*} (t) = V_{o} \;e^{{\left( {\tfrac{{\alpha^{*} }}{\beta }} \right)\left( {1 - e^{ - \beta \,t} } \right)}} ,$$

where

$$\alpha^{*} = \left[ {a_{1} \,\left( {1 - e^{ - \gamma \,t} } \right) + a_{2} } \right]\,\alpha ,$$

with

$$a_{1} = \left( {\frac{i}{{i_{o} }}} \right)\left( {2 - \frac{i}{{i_{o} }}} \right),$$

and

$$a_{2} = \left( {1 - \frac{i}{{i_{o} }}} \right),$$

where V^*^(t), α, β, α^*^, i, i_o_ and γ were used to denote the tumor volume at time t after DC treatment, the intrinsic growth rate of the tumor, the growth deceleration factor related to the endogenous antiangiogenesis processes, the modified tumor growth rate due to EChT application, the DC intensity, the polarization current and the first-order exponential decay rate of the net effect induced in the solid tumor after the DC was removed, respectively. Dimensionless parameters a_1_ and a_2_ depended on the (i/i_o_) ratio [4–6, 14].

### Interpolation of experimental data

Hermite interpolation was used to interpolate volume data for the individual F3II mammary carcinoma in each experimental group. It was advised to know the values of α, β, γ and i_o_ of this equation, as suggested in [5]. This was suggested because the data used were not enough for their fitting with Eq. (1).

### Criteria for model assessment

Values and their estimation accuracies of α, β, γ and i_o_ for the MGE were computed for each mouse by means of following criteria for model assessment [4–6, 14]

$$D_{{{\text{m\'ax}}}} = \text{m\'ax}\left| {F_{i} - \left. {G_{i} } \right|} \right.,$$

$$RMSE = \sqrt {\sum\limits_{i = 1}^{M} {\frac{{\left( {F_{i} - G_{i} } \right)^{2} }}{M}} } ,$$

$$SSE = \sum\limits_{j = 1}^{{n_{1} }} {\left( {\widehat{V}_{j}^{*} - V_{j}^{*} } \right)^{2} } ,$$

$$SE = \sqrt {\frac{{\sum\nolimits_{j = 1}^{{n_{1} }} {\left( {\widehat{V}_{j}^{*} - V_{j}^{*} } \right)^{2} } }}{{n_{1} - k}}} ,$$

$$r_{a}^{2} = 1 - \frac{{n_{1} - 1}}{{n_{1} - k}}\,\left( {1 - r^{2} } \right) = \frac{{\left( {n_{1} - 1} \right)\;r^{2} - k + 1}}{{n_{1} - k}}\,,$$

$$PRESS = \frac{{\sum\nolimits_{j = 1}^{{n_{1} - 1}} {\left[ {\left( {\widehat{V}_{j}^{*} } \right)^{\prime } - V_{j}^{*} } \right]^{2} } }}{{n_{1} - k}},$$

$$MPRESS(m) = \frac{{\sum\nolimits_{j = m + 1}^{{n_{1} }} {\left[ {\left( {\hat{V}_{j}^{*} } \right)^{\prime } - V_{j}^{*} } \right]^{2} } }}{{n_{1} - m}},$$

$$1 - r^{2} = \frac{{\sum\nolimits_{j = 1}^{{n_{1} }} {\left( {\hat{V}_{j}^{*} - V_{j}^{*} } \right)^{2} } }}{{\sum\nolimits_{j = 1}^{{n_{1} }} {\left( {V_{j}^{*} } \right)^{2} - \frac{1}{{n_{1} }}\left( {\sum\nolimits_{j = 1}^{{n_{1} }} {V_{j}^{*} } } \right)^{2} } }},$$

where D_max_, RMSE, SSE, SE, $r^{2}$, $r_{a}^{2}$, PRESS and MPRESS were used to denote the maximum distance, Root Means Square Error, sum of squares of errors, standard error of the estimate, goodness-of-fit, adjusted goodness-of-fit coefficient of multiple determination, predicted residual error sum of squares and multiple predicted residual sum error of squares, respectively. SSE, SE, $r^{2}$, $r_{a}^{2}$, PRESS and MPRESS were chosed as fitting quality criteria. Least Sum of Squares of Errors was obtained when SSE was minimized in the Marquardt–Levenberg optimization algorithm.

RMSE and D_max_ were used to know how different the average TGK of each treated group respect its respective control group. In expressions of D_max_ and RMSE, M, F_i_ and G_i_ were defined as the number of interpolated data of tumor kinetics [tumor volume versus time plot], the *i*-th tumor volume of the control group that was chosen as the reference and the *i*-th tumor volume of the treated group to be compared with the reference group, respectively. Nevertheless, in the expresions of SSE, SE, $r^{2}$, $r_{a}^{2}$, PRESS and MPRESS, $V_{j}^{*}$ was defined as the *j*-th measured tumor volume at discrete time t_j_, j = 1, 2, …, n_1_. $\hat{V}_{j}^{*}$ was denoted as the *j*-th estimated tumor volume by MGE. n_1_ was the number of experimental points (n_1_ = 11). The number of parameters was symbolized by k. The fitting was considered to be satisfactory when $r_{a}^{2}$  > 0.98. A better fit was meant higher $r_{a}^{2}$. $\left( {V_{j}^{*} } \right)^{\prime }$ was designated as the estimated value of $V_{j}^{*}$ when MGE was obtained without the *j*-th observation. MPRESS removed the last $n_{1} - m$ measurements. The model was fitted to the first m measured experimental points (m = 3, 4 or 5) and then from calculated model parameters the error between tumor volume estimated and measured values in the remaining $n_{1} - m$ points was calculated [4–6, 14].

The analysis of the TGK in each experimental group was done following the same procedure reported in [5]. Fitting the full TGK (18–49 days post-inoculation) was performed for CG1-F, CG2-F, CG1-M and CG2-M. For this, each time instant belonging to this range of days was subtracted 18 days so that the Eq. (1) satisfied V^*^(t = 0) = V_o_ (initial condition for V^*^(t)). In this case, V_o_ was the individual F3II mammary carcinoma volume reached at 18 days in each animal of CG1-F, CG2-F, CG1-M and CG2-M. The tumor volume of 0.5 cm^3^ was approximately observed at 31 days post-inoculation (t = 0 for therapy), time in which DC was applied. The analysis per section of TGK was done in two parts (TGK_1_ and TGK_2_) for each animal and gender in all experimental groups. TGK_1_ and TGK_2_ comprised the time intervals from 18 to 31 and 31 to 49 days post-inoculation, respectively. Each time instant in TGK_1_ was subtracted 18 days whereas in TGK_2_ 31 days to fit each one of these two parts. This guaranteed that the MGE fulfilled V^*^(t = 0) = V_o_, where V_o_ was the individual F3II mammary carcinoma volume reached at 18 and 31 days post-inoculation for TGK_1_ and TGK_2_, respectively. Once TGK_1_ was fitted for each individual tumor, the values of α and β were introduced into the MGE to fit TGK_2_, taking into account that the Eq. (1) corresponded with the unperturbed Gompertz equation (DC intensity was equal to zero). This procedure permitted the analysis of the TGK to be conducted in the same mouse before and after EChT application, as suggested in [5].

### Statistical criteria

The random distribution of mice by experimental group was done with the software of random numbers LABTOOLS version 2.0, 1996 (Centro de Investigaciones y Evaluaciones Biológicas, Instituto de Farmacia y Alimentos, La Habana, Cuba). Additionally, statistical analyses were carried out using Minitab 14 statistical software (Minitab for Windows, 2003, free software, National Institute of Standards and Technology, State University of Pensilvania, USA, https://www.minitab.com/es-mx/products/minitabs) for a confidence of 95%. Kolmogorov–Smirnov test was used to determine the normal distribution of parameters. Levene’s test was used to know the homogeneity of variance. Kruskall-Wallis test and two-tailed Mann–Whitney test were applied to compare body mass and tumor volumes between the experimental groups, respectively. Logrank test was used for survival analysis, using free professional software GraphPad Prism (version 5.00, GraphPad Software, San Diego, CA). Both professional software programs worked on a PC (Intel Core i3 processor at 3.3 GHz) located at CENPALAB.

In order to fit each mouse growth curve Eq. (1) was used. A computer program was implemented in the Matlab software (version R2012b 64-bit, University Institute for Research in Mathematics and Applications, University of Zaragoza, Zaragoza, Spain) to calculate the tumor volume. In addition, the mean ± mean standard error of the parameters α, β, γ, i_0_, SSE, SE, $r_{a}^{2}$, PRESS, MPRESS, RMSE and D_max_ were calculated from their individual values. In addition, the estimation errors of the parameters α, β, γ and i_0_, named e_α_, e_β_, e_γ_ and e_i0_, respectively was computed. Mean standard error was calculated as (standard deviation)/ $\sqrt {\text{N}}$, where N was the total number of determinations. These calculations were performed on a PC with an Intel(R) core processor™ i7-3770 at 3.40 GHz with a Windows 10 operating system. All calculations took approximately 10 min. In this study, mm were converted to cm (centimeters).

## Results

### Experimental results

Before the inoculation of tumoral cells, it were computed the average ± mean standard error of the weight of 80 males (22.72 ± 1.03 g) and 80 females (21.27 ± 1.22 g). For 80 female mice were estimated the average ± mean standard error of the latency time of the F3II mammary carcinoma: 15.4 ± 3.3 days for CG1, 13.2 ± 2.2 days for CG2, 12.0 ± 2.1 days for TG1, 11.6 ± 3.6 days for TG2, 13.2 ± 3.2 days for TG3, 12.6 ± 4.1 days for TG4, 14.0 ± 3.8 days for TG5 and 15.0 ± 4.8 days for TG6, respectively. For 80 male mice were calculated the average ± mean standard error of the latency time of the F3II mammary carcinoma: 11.8 ± 4.2 days for CG1, 12.3 ± 3.0 days for CG2, 11.0 ± 2.0 days for TG1, 12.0 ± 1.1 days for TG2, 11.5 ± 2.0 days for TG3, 12.8 ± 4.5 days for TG4, 12.2 ± 3.9 days for TG5 and 11.0 ± 4.4 days for TG6, respectively. Statistically significant differences were not observed between the F3II tumor latency time when experimental groups were compared, according to one tail Mann–Whitney U test (p > 0.05). It was observed that 100% of mice carried a tumor at the time of the DC application.

It was observed that 24.40% (39/160) of all mice died before DC application (31 days after the inoculation): 28.80% (23/80) for females and 20.00 (16/80) for males. These deaths were explained by three main reasons. Firstly, multiple metastasic nodules in lungs (19.38% = 31/160) were observed in CG1-F (3), CG1-M (4), CG2-F (3), CG2-M (4), TG2-F (2), TG3-F (4), TG4-F (1), TG5-F (4), TG5-M (1), TG6-F (3) and TG6-M (2). This finding was confirmed in the histological study of organs. Secondly, animal deaths (2.50% = 4/160) in TG2-F (2), TG2-M (1) and TG4-F (1) were seen after anesthesia administration. Thirdly, euthanasia (2.50% = 4/160) was carried out in mice of TG2-M (2) and TG6-M (2) due to very large tumors (tumor volumes ≥ 2 cm^3^).

**Fig. 2:**
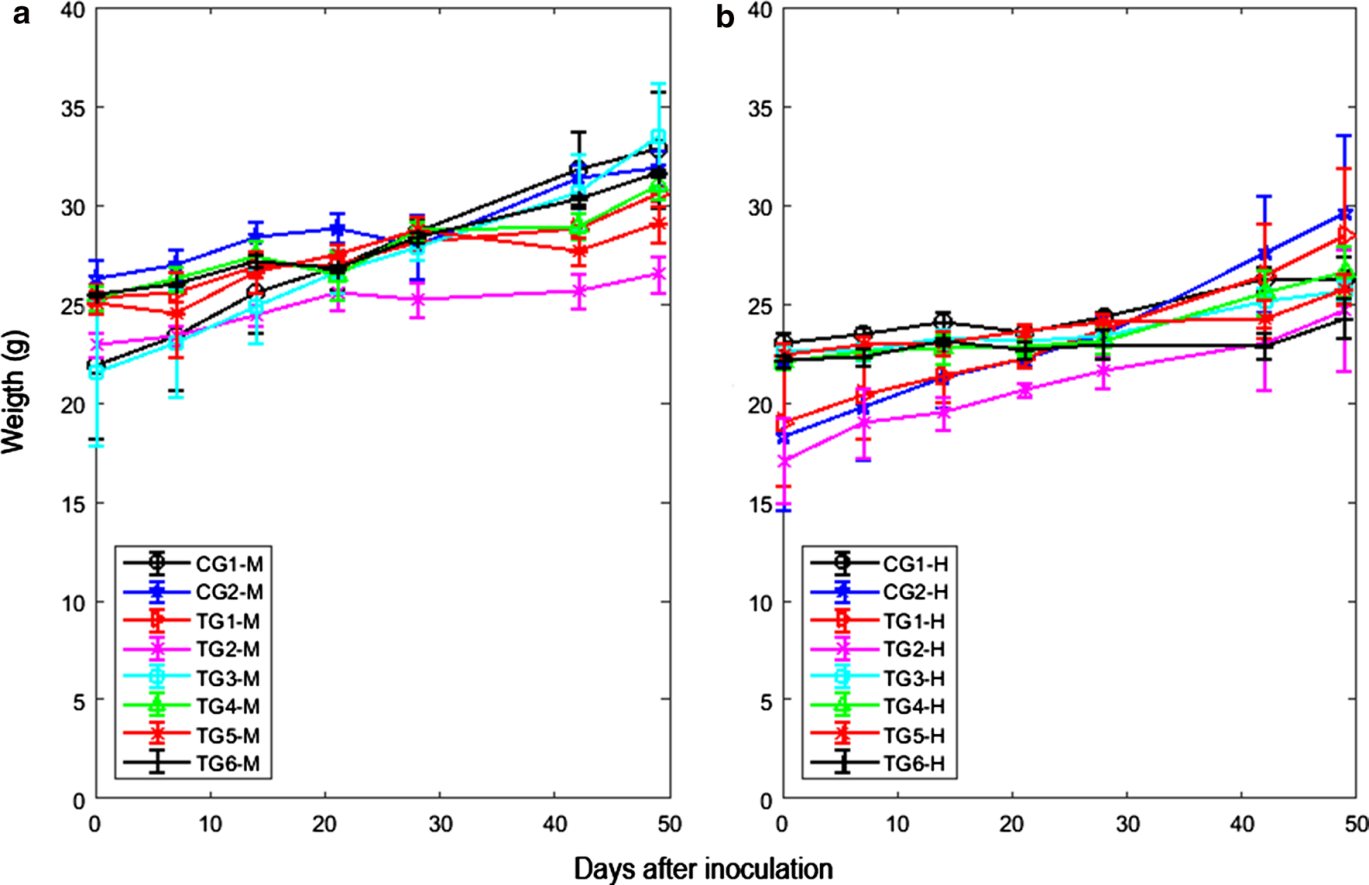
Mean ± mean standard error of corporal weight of the mice BALB/c/Cenp versus days post-inoculation of the tumoral cells F3II. **a** Males. **b** Females. The legends of the experimental groups were defined in Methods

It was calculated the average ± mean standard error of the mice weight for 64 males (25.59 ± 1.05 g) and 57 females (23.31 ± 1.06 g) before DC application. The weight of male mice was significantly higher than that female mice (Fig. 2). Except for TG2 in both genders, statistically significant differences between weights of male and female mice, in each experimental group, were not observed (p > 0.05). In this group, from the beginning of the experiment, the weight was significantly lower, with regard to the rest of the experimental groups (p = 0.0012), according to the statistical evaluation for ANOVA test (Fig. 2).

It was reported that 13.48% (19/141) of mice died after EChT application. These deaths were observed in 84.21% (16/19) of mice (six females and 10 males) at the first 72 h after DC application and 15.79% (3/19) after 72 h post-treatment (two females and one male). All these deaths were confirmed by histological study of organs.

At the first 72 h after DC application, deaths were observed in six mice of control groups (two of CG1-F, one of CG1-M, two of CG2-F and one of CG2-M) and five mice of DC treated groups (one of TG2-M, one of TG3-M, one of TG4-F, one of TG5-M and one of TG6-F) due to multiple metastatic nodules in lungs. Other five mice deaths were documented by alterations in their organs: heart damage (mechanical rupture by insertion of electrodes pair) was observed in one mouse of TG5-M, liver damage was seen in one mouse of TG2-M and damages in both liver and kidney organs were perceived in one mouse of TG3-M and two mice of TG4-M. Additionally, deaths by lung metastasis (one mouse of TG3-F and one mouse of TG5-F) and damages in both liver and kidney organs (one mouse of TG6-M) were reported 72 h post-treatment.

As a result of deaths mentioned-above, all experimental results for 76.250% (122/160) of mice were reported in this study: 66.25% (53/80) of males and 61.25% (49/80) of females. The gain of corporal weight of mice BALB/c/Cenp was shown in Table 1 for days 7, 14, 21, 28, 42 and 49 after inoculation, by experimental group and gender. In this table was revealed that there was not exist statistically significant differences (p > 0.05) when the treated groups were compared with their respective groups control, for each gender, according to ANOVA test.

**Table 1:** Mean ± mean standard error of the body weight gain of mice after direc current application by group experimental and gender

Groups	G	Gain of corporal weight (in g)					
		7	14	21	28	42	49
CG1	M (N = 5)	0.62 ± 0.29	1.96 ± 0.36	2.38 ± 0.20	3.22 ± 0.43	4.58 ± 0.88	4.60 ± 1.22
	F (N = 5)	0.42 ± 0.18	1.07 ± 0.59	0.59 ± 0.50	1.34 ± 0.47	3.30 ± 1.03	3.30 ± 1.55
CG2	M (N = 5)	0.72 ± 0.55	2.10 ± 0.80	2.56 ± 0.64	1.63 ± 1.70	5.04 ± 0.43	5.60 ± 0.94
	F (N = 5)	0.38 ± 0.24	0.80 ± 0.49	0.60 ± 0.38	0.64 ± 0.22	2.75 ± 0.72	3.84 ± 0.88
TG1	M (N = 10)	0.30 ± 0.51	1.62 ± 0.42	1.64 ± 0.43	2.78 ± 0.57	3.50 ± 0.74	5.29 ± 0.54
	F (N = 10)	0.55 ± 0.19	0.44 ± 0.36	0.44 ± 0.36	0.93 ± 0.20	1.72 ± 0.52	2.88 ± 0.34
TG2	M (N = 5)	0.45 ± 0.43	1.46 ± 0.53	2.66 ± 0.65	2.24 ± 0.77	2.67 ± 0.44	3.53 ± 0.45
	F (N = 6)	1.29 ± 0.16	0.95 ± 0.12	1.40 ± 0.17	1.57 ± 0.20	1.47 ± 0.18	2.47 ± 0.31
TG3	M (N = 8)	0.61 ± 0.47	1.64 ± 0.14	2.52 ± 0.77	2.75 ± 0.25	3.65 ± 0.46	5.83 ± 0.46
	F (N = 5	− 0.07 ± 0.42	0.60 ± 0.35	0.47 ± 0.34	0.75 ± 0.43	2.53 ± 0.43	3.05 ± 0.34
TG4	M (N = 8)	0.99 ± 0.13	2.12 ± 0.40	1.12 ± 1.15	3.43 ± 0.53	3.62 ± 0.77	5.70 ± 0.86
	F (N = 7)	0.49 ± 0.15	0.66 ± 0.48	0.75 ± 0.16	1.05 ± 0.37	3.50 ± 0.93	4.59 ± 1.09
TG5	M (N = 7)	− 0.59 ± 1.82	1.54 ± 0.63	2.41 ± 0.32	3.68 ± 0.43	2.60 ± 0.46	3.97 ± 0.41
	F (N = 5)	0.50 ± 0.24	0.62 ± 0.35	1.29 ± 0.31	1.65 ± 0.52	1.87 ± 1.02	3.37 ± 0.74
TG6	M (N = 5)	0.51 ± 0.03	1.72 ± 0.27	1.30 ± 0.24	2.90 ± 0.18	4.81 ± 0.29	6.17 ± 0.25
	F (N = 6)	0.20 ± 0.24	0.95 ± 0.27	0.53 ± 0.27	0.83 ± 0.60	0.94 ± 0.46	2.13 ± 1.22

G, N, F and M were identified as the gender, number of mice by experimental group and gender, female gender and male gender, respectively. The legend of each experimental group was defined in the topic Methods

The mean ± mean standard error of the tumor regression percentages were documented for each experimental group and gender: 62.33 ± 28.45% for TG1-F (N = 10), 70.67 ± 25.48% for TG1-M (N = 10), 74.00 ± 26.16% for TG2-F (N = 6), 77.43 ± 28.08% for TG2-M (N = 5), 44.50 ± 23.44% for TG3-F (N = 5), 61.75 ± 27.72% for TG3-M (N = 8), 36.33 ± 29.54% for TG4-F (N = 7), 45.67 ± 28.62% for TG4-F (N = 8), 30.33 ± 27.77% for TG5-F (N = 5), 37.33 ± 21.42% for TG5-M (N = 7), 61.00 ± 29.90% for TG6-F (N = 6) and 57.33 ± 29.43% for TG6-M (N = 5). The highest regression percentages were observed for TG2-F and TG2-M.

The quantity of DC treated F3II mammary carcinomas distributed for each tumor response type (PD, SD, PR or CR) and EChT global effectiveness were displayed in Table 2, by experimental group and gender. The higher effectiveness of the DC was observed for the TG2-F (66.6%) and TG2-M groups (80%) and the lowest for both genders in the TG4 and TG5 groups. Animals that died before treatment were not included in these percentages. The tumor remission was not observed in the tumors of CG1-F, CG2-F, CG1-M and CG2-M, which ruled out the induction of spontaneous remissions of F3II carcinoma.

**Table 2:** Different F3II tumor response types after electrochemical therapy application by experimental group and gender

Groups	Gender	F3II tumor responses after EChT application				Overall effectiveness (%) (PR + CR)
		PD (%)	SD (%)	PR (%)	CR (%)	
TG1	F (N = 10)	1 (10.0)	4 (40.0)	5 (50.0)	0 (0.0)	5 (50.0)
	M (N = 10)	1 (10.0)	3 (30.0)	6 (60.0)	0 (0.0)	6 (60.0)
TG2	F (N = 6)	1 (16.7)	1 (16.7)	4 (66.6)	0 (0.0)	4 (66.6)
	M (N = 5)	0 (0.0)	1 (20.0)	4 (80.0)	0 (0.0)	4 (80.0)
TG3	F (N = 5)	1 (20.0)	1 (10.0)	3 (30.0)	0 (0.0)	3 (30.0)
	M (N = 8)	2 (25.0)	3 (25.0)	4 (50.0)	0 (0.0)	4 (50.0)
TG4	F (N = 7)	2 (28.6)	3 (42.8)	2 (28.6)	0 (0.0)	2 (28.6)
	M (N = 8)	2 (25.0)	3 (37.5)	4 (50.0)	0 (0.0)	4 (50.0)
TG5	F (N = 5)	1 (20.0)	1 (20.0)	2 (40.0)	0 (0.0)	2 (40.0)
	M (N = 7)	1 (14.4)	3 (42.8)	3 (42.8)	0 (0.0)	3 (42.8)
TG6	F (N = 6)	1 (16.7)	2 (33.3)	3 (50.0)	0 (0.0)	3 (50.0)
	M (N = 5)	1 (20.0)	2 (40.0)	2 (40.0)	0 (0.0)	2 (40.0)

N was defined as the total number of mice carrying the F3II mammary carcinoma in each experimental group. The percentage (in brackets) by tumor response after electrochemical therapy was represented by  %. PD, SD, PR and CR were denoted as progressive disease, stable disease, partial response and complete response, respectively. The legend of each experimental group was defined in the topic Methods

The mean ± standard error of the doubling time of the F3II mammary carcinoma (in days) were presented in Table 3 by each gender and experimental group. It was also observed that in the control groups of both genders, this kinetics parameter was lower than that in the treated groups. Additionally, an increase in the doubling time of this malignat tumor of all treated groups was induced by DC action, except in TG4-F. This increase was statistically significant (p = 0.0497) in the TG2-F, TG2-M, TG3-F and TG6-F groups, according to the one-tail Mann–Whitney U test.

**Table 3:** Mean ± mean standard error of the tumor doubling time before and after 31 days post-inoculation by experimental group and gender

Groups	Gender	Doubling time of the F3II mammary carcinoma (days)	
		Before DC treatment	After DC treatment
CG1	M (N = 5)	4.33 ± 0.92	6.00 ± 2.20
	F (N = 5)	4.27 ± 1.97	6.50 ± 1.06
CG2	M (N = 5)	3.60 ± 0.93	3.20 ± 0.86
	F (N = 5)	2.00 ± 1.51	4.80 ± 1.02
TG1	M (N = 10)	1.60 ± 0.59	5.60 ± 1.80
	F (N = 10)	4.25 ± 1.22	7.00 ± 1.18
TG2	M (N = 5)	7.20 ± 2.35	11.20 ± 2.95^a^
	F (N = 6)	5.17 ± 0.83	12.75 ± 2.35^a^
TG3	M (N = 8)	5.17 ± 1.28	7.00 ± 1.93
	F (N = 5	3.75 ± 1.25	9.25 ± 2.02^a^
TG4	M (N = 8)	5.40 ± 1.72	6.20 ± 2.45
	F (N = 7)	5.80 ± 1.24	3.80 ± 0.58
TG5	M (N = 7)	3.80 ± 0.37	5.40 ± 1.16
	F (N = 5)	3.75 ± 1.38	5.33 ± 1.45
TG6	M (N = 5)	3.50 ± 2.51	5. 66 ± 4.01
	F (N = 6)	5.00 ± 1.45	15.40 ± 0.98^a^

The number of mice by gender in each experimental, female gender and male gender were represented by N, F and M, respectively. ^a^Statistically significant differences (p = 0.0497) according to one tail Mann–Whitney U test. The legend of each experimental group was defined in the topic Methods

On the other hand, the values of tumor growth delay were computed for TG1-F (1.08 days with N = 100), TG1-M (0.93 with N = 10), TG2-F (1.82 days with N = 6), TG2-M (2.00 days; N = 5), TG3-F (1.93 days; N = 5), TG3-M (2.19 days; N = 8), TG4-F (0.79 days; N = 7), TG4-M (1.94 days; N = 8), TG5-F (1.11 days; N = 5), TG5-M (1.69 days; N = 7), TG6-F (3.21 days; N = 6) and TG6-M (1.77 days; N = 5). The higher tumor growth delay was observed in TG2-F and TG2-M when MSNEII_c_ was used. Additionally, the higher tumor growth delays were seen in TG6-F and TG3-M, being noticeable for TG3-M.

Physiological ulcerations were observed in the tumors of the control groups and DC treated groups. Furthermore, fibrosis of hard texture (with aspect of scab) were observed after EChT applicaction in some tumors of each treated group.

**Fig. 3:**
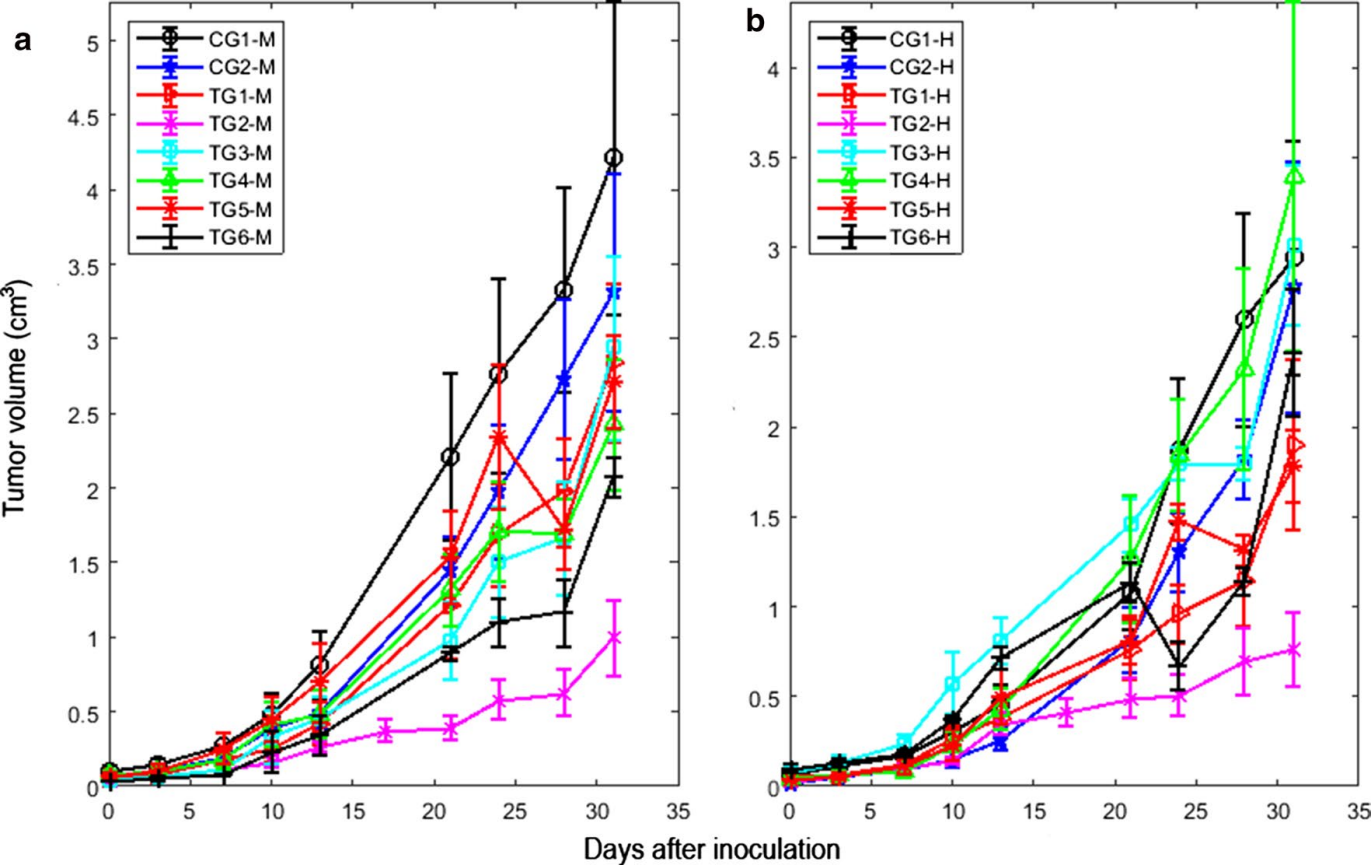
Mean ± mean standard error of the tumor volume against post-inoculation days of F3II tumor cells. **a** Males BALB/c/Cenp mice (M). **b** Females BALB/c/Cenp mice (F). Legends of each experimental group were defined in the Experiment subsection

In Fig. 3 was showed the temporal behavior of the tumor volume after the inoculation of the tumor cells in the host by each experimental group for males (Fig. 3a) and females (Fig. 3b). The growth of primary F3II mammary carcinoma of the CG1-F and CG2-F was greater than that of the CG1-M and CG2-M, respectively. This finding was explained in [5]. In addition, a delay in the growth of this tumor histological variety was observed in each treated group with respect to that of their respective control groups for females and males.

**Fig. 4:**
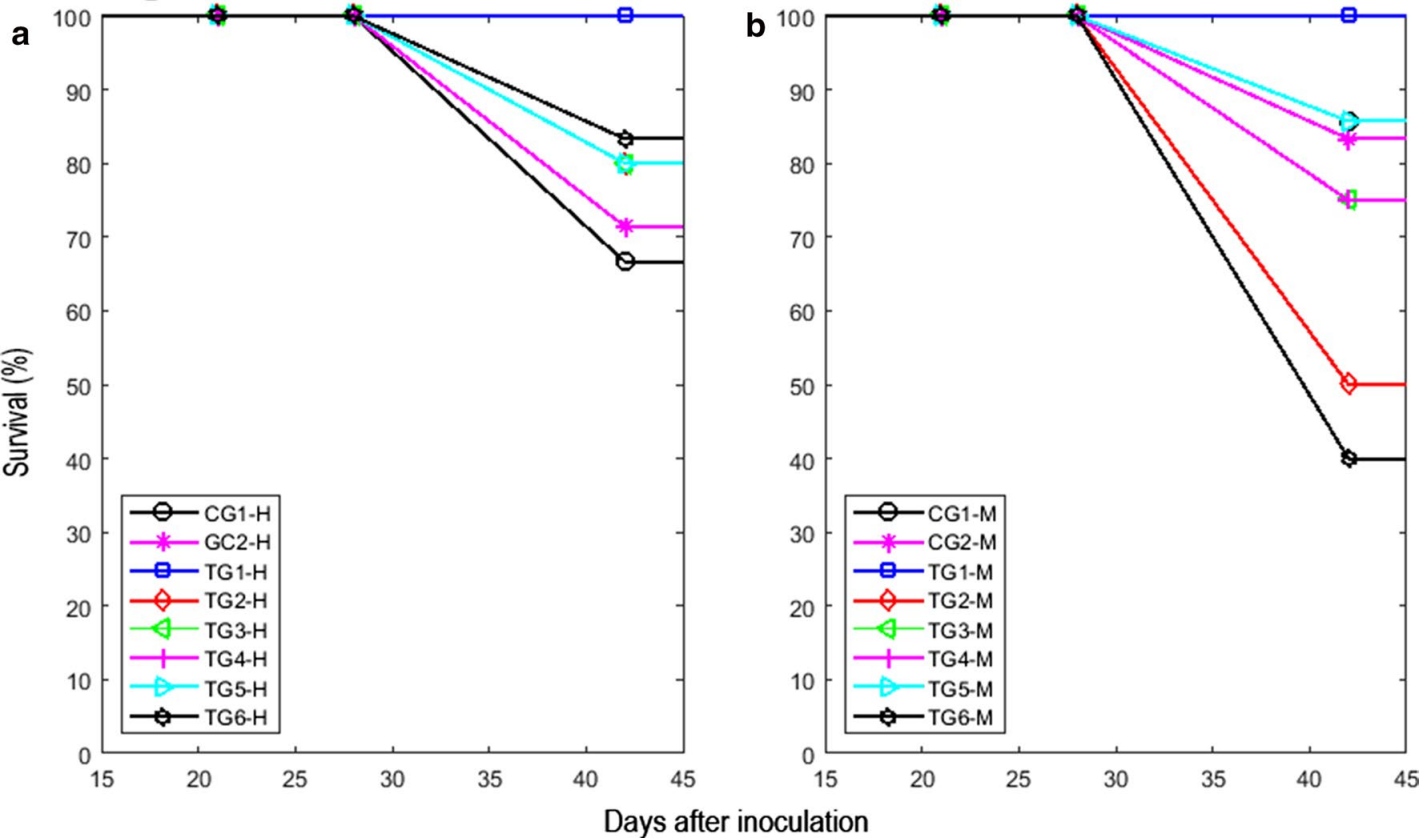
Survival versus days after inoculation of F3II cells in mice BALB/c/Cenp, by experimental group and gender. **a** Females. **b** Males. CG1 (first control group, in which C-I was used but DC was not applied). CG2 (second control group, in which C-II was used but DC was not applied). TG1 (first group treated with 2 mA for 10 min and C-I). TG2 (second group treated with 6 mA for 20 min and C-II). TG3 (third group treated with 2 mA for 10 min and C-III). TG4 (fourth group treated with 2 mA for 10 min and C-II). TG5 (fifth group treated with 6 mA for 10 min and C-II). TG6 (sixth group treated with 10 mA for 10 min and C-II). C-I was formed by four collinear electrodes with alternating polarities inserted perpendicularly along the largest diameter of the tumor (electrodes 1 and 3 were positives whereas electrodes 2 and 4 negatives), as shown in Fig. 1a. C-II were formed by four electrode pairs inserted into the tumor to 45° (electrode pair 1–2), 135° (electrode pair 3–4), 225° (electrode pair 5–6) and 325° (electrode pair 7–8), as shown in Fig. 1b. For C-II, electrodes 1, 4, 5 and 8 were positives whereas electrodes 2, 3, 6 and 7 negatives. C-III were formed by four electrode pairs arranged into the tumor to 0° (electrode pair 1–2), 90° (electrode pair 3–4), 180° (electrode pair 5–6) and 270° (electrode pair 7–8), as shown in Fig. 1c. For C-III, electrodes 2, 3, 5 and 8 were positives whereas electrodes 1, 4, 6 and 7 negatives

In Fig. 4 was displayed the overall survival percentages of females (Fig. 4a) and males (Fig. 4b) for each experimental group. A significant decrease in the overall survival of the CG1-F mice with respect to TG1-F (p = 0.0142) was documented when the Logrank test was used. In addition, it was reported a significant decrease of this parameter in the TG2-M and TG6-M groups (p = 0.0321) when compared to CG1-M and CG2-M, respectively. Additionally, the mice survival in TG2-M lower than that in CG1-M was revealed. This finding was also observed in TG3-M, TG4-M and TG6-M with respect to CG2-M. 100% of the mice survival in TG1-M and TG1-F was reported.

Very larger tumor volumes (above 3 cm^3^) at 49 days after inoculation were observed in the majority of mice in all experimental group, except in TG2-F and TG2-M. Consequently, all mice were sacrified in order to perform ethical aspects in laboratory animals (tumor burden did not exceed 10% of the host animal’s normal body weight), as reported in [5, 11].

### Analysis of interpolated data

Mathematical modeling was suggested to analyse TGK once finished the experimental part of this study. Although in Fig. 3 were shown larger mean standard errors of tumor volumes, fitting TGK of 21 (21/122 = 17.21%) mice (eight females and 13 males) were not made because their tumor volumes were higher and equal than 2 cm^3^, taken into account ethical aspects in laboratory animals above-mentioned. Consequently, 80 BALB/c/Cenp mice (40 males and 40 females) were included to fit individual TGK. Each experimental group was formed by 5 females and 5 males.

The averages and mean standard errors of α, β, e_α_, e_β_, SSE, SE, PRESS, MPRESS, RMSE and D_max_ obtained from the individual analysis of TGK_1_, by experimental group, were shown in Tables 4 and 5 for females and males, respectively. It was reported that females showed higher values α and lower β than those for males, which corroborated that TGK was faster in females than in males, as in [5]. Additionally, α^*^ decreased respect to α in all DC treated groups. Nevertheless, β did not change significantly before and after DC application (Tables 4 and 5).

**Table 4:** Mean ± mean standard error of parameters obtained from fitting of TGK_1_ for female groups

Parameters	Experimental groups							
	CG1-F	CG2-F	TG1-F	TG2-F	TG3-F	TG4-F	TG5-F	TG6-F
α (days^−1^)	0.462 ± 0.044	0.490 ± 0.189	0.462 ± 0.044	0.490 ± 0.189	0.490 ± 0.189	0.490 ± 0.189	0.490 ± 0.189	0.490 ± 0.189
α^*^ (days^−1^)	–	–	0.167 ± 0.019	0.300 ± 0.057	0.224 ± 0.033	0.197 ± 0.027	0.206 ± 0.014	0.187 ± 0.049
β (days^−1^)	0.024 ± 0.026	0.013 ± 0.060	0.009 ± 0.005	0.055 ± 0.029	0.026 ± 0.014	0.023 ± 0.004	0.001 ± 0.001	0.016 ± 0.016
e_α_ (days^−1^)	0.011 ± 0.003	0.008 ± 0.070	0.098 ± 0.075	0.073 ± 0.061	0.012 ± 0.005	0.015 ± 0.007	0.089 ± 0.057	0.037 ± 0.016
e_β_ (days^−1^)	0.003 ± 0,000^*^	0.007 ± 0.002	0.006 ± 0.001	0.051 ± 0.006	0.001 ± 0.000^**^	0.008 ± 0.001	0.009 ± 0.003	0.015 ± 0.001
SSE (cm^3^)	0.016 ± 0.007	0.011 ± 0.002	0.113 ± 0.032	0.154 ± 0.051	0.613 ± 0.493	0.222 ± 0.107	0.079 ± 0.026	0.712 ± 0.248
SE (cm^3^)	0.019 ± 0.005	0.017 ± 0.001	0.026 ± 0.003	0.029 ± 0.005	0.048 ± 0.024	0.033 ± 0.009	0.022 ± 0.004	0.061 ± 0.015
$r_{a}^{2}$	0.994 ± 0.002	0.964 ± 0.015	0.977 ± 0.006	0.956 ± 0.007	0.980 ± 0.010	0.938 ± 0.038	0.964 ± 0.019	0.958 ± 0.013
PRESS (cm^3^)	0.001 ± 0.001	0.002 ± 0.001	0.012 ± 0.003	0.018 ± 0.006	0.029 ± 0.018	0.035 ± 0.022	0.016 ± 0.012	0.149 ± 0.063
MPRESS (cm^3^)	0.001 ± 0.001	0.002 ± 0.001	0.012 ± 0.003	0.018 ± 0.006	0.029 ± 0.018	0.035 ± 0.023	0.016 ± 0.012	0.151 ± 0.064
RMSE (cm^3^)	0.018 ± 0.005	0.017 ± 0.002	0.026 ± 0.003	0.029 ± 0.005	0.048 ± 0.024	0.032 ± 0.009	0.022 ± 0.004	0.060 ± 0.015
D_max_ (cm^3^)	0.034 ± 0.010	0.038 ± 0.010	0.054 ± 0.008	0.059 ± 0.011	0.110 ± 0.047	0.074 ± 0.023	0.054 ± 0.004	0.118 ± 0.031

^*^0.0031 ± 0.0002,^**^0.0012 ± 0.0001. TGK_1_ was the first part of the unperturbed and direct current perturbed F3II tumor growth kinetic. The variable α was the intrinsic growth rate of the tumor and e_α_ its estimation error. The variable β was the growth decelation factor and e_β_ its estimation error. The maximum distance, root means square error, sum of squares of errors, standard error of the estimate, adjusted goodness-of-fit coefficient of multiple determination, predicted residual error sum of squares and multiple predicted residual sum error of squares parameters were represented by D_max_, RMSE, SSE, SE, $r_{a}^{2}$, PRESS and MPRESS (for m = 3), respectively. Legends of CG1-F, CG2-F, TG1-F, TG2-F, TG3-F, TG4-F, TG5-F and TG6-F were defined in Experiment subsection

**Table 5:** Average ± mean standard error of parameters obtained from fitting of TGK_1_ for male groups

Parameters	Experimental groups							
	CG1-M	CG2-M	TG1-M	TG2-M	TG3-M	TG4-M	TG5-M	TG6-M
α (days^−1^)	0.426 ± 0.059	0.422 ± 0.031	0.426 ± 0.059	0.422 ± 0.031	0.422 ± 0.031	0.422 ± 0.031	0.422 ± 0.031	0.422 ± 0.031
α^*^ (days^−1^)	–	–	0.226 ± 0.107	0.303 ± 0.051	0.201 ± 0.046	0.174 ± 0.049	0.244 ± 0.034	0.202 ± 0.087
β (days^−1^)	0.034 ± 0.022	0.049 ± 0.016	0.059 ± 0.044	0.078 ± 0.050	0.017 ± 0.017	0.058 ± 0.046	0.028 ± 0.010	0.014 ± 0.014
e_α_ (days^−1^)	0.021 ± 0.050	0.011 ± 0.070	0.098 ± 0.057	0.037 ± 0.061	0.010 ± 0.003	0.015 ± 0.007	0.089 ± 0.057	0.037 ± 0.016
e_β_ (days^−1^)	0.001 ± 0.000	0.007 ± 0.000^*^	0.002 ± 0.001	0.015 ± 0.002	0.002 ± 0.000^**^	0.009 ± 0.001	0.009 ± 0.003	0.051 ± 0.008
SSE (cm^3^)	0.184 ± 0.068	0.055 ± 0.032	0.147 ± 0.079	0.053 ± 0.014	0.300 ± 0.238	0.334 ± 0.219	0.072 ± 0.023	0.138 ± 0.095
SE (cm^3^)	0.064 ± 0.016	0.033 ± 0.011	0.026 ± 0.008	0.017 ± 0.003	0.063 ± 0.032	0.036 ± 0.015	0.019 ± 0.004	0.028 ± 0.011
$r_{a}^{2}$	0.979 ± 0.003	0.985 ± 0.007	0.964 ± 0.016	0.980 ± 0.005	0.922 ± 0.019	0.978 ± 0.008	0.991 ± 0.005	0.965 ± 0.007
PRESS (cm^3^)	0.030 ± 0.016	0.004 ± 0.002	0.023 ± 0.019	0.003 ± 0.001	0.018 ± 0.010	0.036 ± 0.020	0.007 ± 0.003	0.004 ± 0.003
MPRESS (cm^3^)	0.030 ± 0.016	0.004 ± 0.002	0.023 ± 0.019	0.003 ± 0.001	0.018 ± 0.010	0.036 ± 0.020	0.007 ± 0.003	0.005 ± 0.003
RMSE (cm^3^)	0.061 ± 0.015	0.032 ± 0.011	0.026 ± 0.008	0.017 ± 0.003	0.061 ± 0.031	0.036 ± 0.015	0.019 ± 0.004	0.028 ± 0.011
D_max_ (cm^3^)	0.137 ± 0.037	0.064 ± 0.023	0.061 ± 0.020	0.035 ± 0.008	0.139 ± 0.065	0.069 ± 0.027	0.036 ± 0.008	0.065 ± 0.009

^*^0.0075±0.0006, ^**^0.0021 ± 0.0003. TGK_1_ was the first part of the unperturbed and direct current perturbed F3II tumor growth kinetic. The variable α was the intrinsic growth rate of the tumor and e_α_ its estimation error. The variable β was the growth decelation factor and e_β_ its estimation error. The maximum distance, root means square error, sum of squares of errors, standard error of the estimate, adjusted goodness-of-fit coefficient of multiple determination, predicted residual error sum of squares and multiple predicted residual sum error of squares were represented by parameters D_max_, RMSE, SSE, SE, $r_{a}^{2}$, PRESS and MPRESS (for m = 3), respectively. Legends of CG1-F, CG2-F, TG1-F, TG2-F, TG3-F, TG4-F, TG5-F and TG6-F were defined in Experiment subsection

In Table 6 was showed the averages and mean standard errors of i_0_, γ, e_i0_, e_γ_, SSE, SE, $r_{a}^{2}$, PRESS, MPRESS, RMSE and D_max_ obtained from the individual analysis of TGK_2_, for females, by experimental group. These values were also shown for males and each group (Table 7). In addition, the values of the (i/i_0_) ratio were shown in Tables 6 and 7.

**Table 6:** Mean ± mean standard error of parameters obtained from fitting of TGK_2_ for direct current treated female groups

Parameters	Experimental groups					
	TG1-F	TG2-F	TG3-F	TG4-F	TG5-F	TG6-F
i_0_ (mA)	1.561 ± 0.057	4.061 ± 0.238	1.413 ± 0.048	1.569 ± 0.062	4.415 ± 0.299	6.942 ± 0.485
i/i_0_	1.333	1.477	1.415	1.275	1.359	1.440
γ (days^−1^)	17.742 ± 6.769	12.890 ± 4.379	26.291 ± 11.321	36.567 ± 8.088	49.672 ± 18.227	13.672 ± 5.227
e_i0_ (days^−1^)	0.016 ± 0.000^*^	0.043 ± 0.004	0.054 ± 0.000^**^	0.093 ± 0.000^***^	0.021 ± 0.006	0.034 ± 0.003
e_γ_ (days^−1^)	0.056 ± 0.002	0.004 ± 0.000^+^	0.921 ± 0.083	0.015 ± 0.004	0.023 ± 0.005	0.007 ± 0.000^++^
SSE (cm^3^)	0.115 ± 0.052	0.068 ± 0.024	0.522 ± 0.268	0.392 ± 0.130	0.226 ± 0.081	0.226 ± 0.081
SE (cm^3^)	0.040 ± 0.013	0.036 ± 0.009	0.082 ± 0.034	0.087 ± 0.017	0.068 ± 0.010	0.068 ± 0.010
RMSE (cm^3^)	0.039 ± 0.013	0.034 ± 0.008	0.080 ± 0.033	0.084 ± 0.017	0.065 ± 0.010	0.065 ± 0.010
PRESS (cm^3^)	0.017 ± 0.013	0.014 ± 0.007	0.103 ± 0.072	0.098 ± 0.048	0.021 ± 0.008	0.021 ± 0.008
MPRESS (cm^3^)	0.017 ± 0.013	0.013 ± 0.007	0.104 ± 0.072	0.098 ± 0.049	0.020 ± 0.007	0.020 ± 0.007
$r_{a}^{2}$	0.997 ± 0.001	0.989 ± 0.006	0.996 ± 0.002	0.991 ± 0.004	0.994 ± 0.002	0.994 ± 0.002
D_max_ (cm^3^)	0.085 ± 0.032	0.065 ± 0.015	0.182 ± 0.068	0.175 ± 0.030	0.158 ± 0.043	0.158 ± 0.043

^*^0.016 ± 0.0001, ^**^0.054 ± 0.0005, ^***^0.093 ± 0.0002, ^+^0.004 ± 0.0001 and ^++^0.007 ± 0.0006. TGK_2_ was the second part of the direct current perturbed F3II tumor growth kinetic. The variable i_0_ was the polarization current and e_i0_ its estimation error. The variable γ was the exponential decay ratio and e_γ_ its estimation error. The maximum distance, root means square error, sum of squares of errors, standard error of the estimate, adjusted goodness-of-fit coefficient of multiple determination, predicted residual error sum of squares and multiple predicted residual sum error of squares were identified by parameters D_max_, RMSE, SSE, SE, $r_{a}^{2}$, PRESS and MPRESS (for m = 3), respectively. Legends of TG1-F, TG2-F, TG3-F, TG4-F, TG5-F and TG6-F were defined in Experiment subsection

**Table 7:** Mean ± mean standard error of parameters obtained from fitting of TGK_2_ for direct current treated male groups

Parameters	Experimental groups					
	TG1-M	TG2-M	TG3-M	TG4-M	TG5-M	TG6-M
i_0_ (mA)	1842 ± 0.158	4.147 ± 0.186	1.495 ± 0.090	1.516 ± 0.075	4.333 ± 0.232	7.119 ± 1.084
i/i_0_	1.086	1.447	0.968	1.319	1.385	1.405
γ (days^−1^)	13.197 ± 3.920	4.536 ± 0.769	7.526 ± 2.513	12.742 ± 6.769	27.890 ± 9.379	10.291 ± 2.321
e_i0_ (days^−1^)	0.021 ± 0.003	0.032 ± 0.004	0.019 ± 0.000^*^	0.013 ± 0.000^**^	0.053 ± 0.009	0.094 ± 0.005
e_γ_ (days^−1^)	0.009 ± 0.002	0.007 ± 0.000^+^	0.008 ± 0.002	0.014 ± 0.009	0.256 ± 0.078	0.048 ± 0.007
SSE (cm^3^)	0.649 ± 0.131	0.101 ± 0.033	0.221 ± 0.079	0.115 ± 0.052	0.068 ± 0.024	0.522 ± 0.268
SE (cm^3^)	0.120 ± 0.003	0.041 ± 0.008	0.068 ± 0.013	0.040 ± 0.013	0.036 ± 0.009	0.082 ± 0.034
RMSE (cm^3^)	0.116 ± 0.004	0.040 ± 0.008	0.066 ± 0.012	0.039 ± 0.013	0.034 ± 0.008	0.080 ± 0.033
PRESS (cm^3^)	0.095 ± 0.040	0.008 ± 0.005	0.031 ± 0.012	0.017 ± 0.013	0.014 ± 0.007	0.103 ± 0.072
MPRESS (cm^3^)	0.095 ± 0.041	0.009 ± 0.005	0.028 ± 0.012	0.017 ± 0.013	0.013 ± 0.007	0.104 ± 0.072
$r_{a}^{2}$	0.960 ± 0.035	0.794 ± 0.198	0.993 ± 0.003	0.997 ± 0.001	0.989 ± 0.006	0.996 ± 0.002
D_max_ (cm^3^)	0.956 ± 0.038	0.992 ± 0.002	0.992 ± 0.003	0.085 ± 0.032	0.065 ± 0.015	0.182 ± 0.068

^*^0.019±0.0005, ^**^0.013±0.0003 and ^+^0.007±0.0001. TGK_2_ was the second part of the direct current perturbed F3II tumor growth kinetic. The variable i_0_ was the polarization current and e_i0_ its estimation error. The variable γ was the exponential decay ratio and e_γ_ its estimation error. The maximum distance, root means square error, sum of squares of errors, standard error of the estimate, adjusted goodness-of-fit coefficient of multiple determination, predicted residual error sum of squares and multiple predicted residual sum error of squares were denoted by parameters D_max_, RMSE, SSE, SE, $r_{a}^{2}$, PRESS and MPRESS (for m = 3), respectively. Legends of TG1-M, TG2-M, TG3-M, TG4-M, TG5-M and TG6-M were defined in Experiment subsection

For female, the higher values of the (i/i_0_) ratio were observed in TG2-F, TG6-F and TG3-F whereas the lower values of γ in TG2-F and TG6-F. These findings were noticeable for TG2-F (Table 6). For male, the higher values of the (i/i_0_) ratio were observed in TG2-M and TG6-M whereas the lower value of γ in TG2-M. These findings were noticeable for TG2-M (Table 7).

Tables 4, 5, 6, 7 showed that the average values of $r_{a}^{2}$ were close to one and the values of SSE, SE, PRESS, MPRESS (for m = 3), RMSE and D_max_ were close to zero. MPRESS values for m = 3, 4 and 5 were similar in each experimental group and for each gender, as in [4, 5]. Therefore, in this study, these MPRESS values were reported for m = 3.

## Discussion

The minus sign of the weight gain in TG3-F and TG5-M at 7 days post-inoculation is interpreted as a decrease of this variable. Nevertheless, weight gain in these two groups increases after 7 days. Non significant differences in weight gain suggests that anesthesia, electrode insertion and applied treatment do not affect this parameter in BALB/c/Cenp mice carrying the F3II carcinoma for both genders.

Different findings of this study corroborate those reported in [5], such as male mice weights higher than those female mice; the weight gain over time of all BALB/c/Cenp mice; the variability of the F3II mammary carcinoma latency time; BALB/c/Cenp mouse suitables for this tumor histological variety (tumor grows in 100% of BALB/c/Cenp mice); cell line highly invasive and metastatic and greater growth delay. Regression percentage and doubling time of this DC treated tumor type.

The greatest growth delay, mean doubling time, regression percentage of the F3II carcinoma and DC overall effectiveness in TG2-F and TG2-M confirm that this tumor histological variety should be treated with low DC intensities and longer exposure time, as suggest in [5]. These findings may suggest that in TG2-F and TG2-M are induced higher tumor damage percentages due to DC cytotoxic action, as reported experimentally in [1–3, 5, 15]. DC cytotoxic action is explained by induction of toxic products into the tumor from the electrochemical reactions around the electrodes during its application [1–3, 16–18] and the immune system activation [5].

High tumor damage percentages by DC action supposes that ϕ (by apoptosis and/or necrosis induced around electrodes) is increased and GF is decreased, where ϕ and GF are cell loss factors and tumor growth fraction, respectively. A a result, the tumor mean doubling time (DT) increases, according to the Steel equation ($DT = {{T_{c} \,\ln 2} \mathord{\left/ {\vphantom {{T_{c} \,\ln 2} {(1 - \varphi )\ln (1 + GF)}}} \right. \kern-0pt} {(1 - \varphi )\ln (1 + GF)}}$) with $GF = {{N_{cc} } \mathord{\left/ {\vphantom {{N_{cc} } {(N_{cc} + N_{n - cc} )}}} \right. \kern-0pt} {(N_{cc} + N_{n - cc} )}}$, where T_c_, N_cc_, and N_n-cc_ are defined the cell cycle time, number of tumor cells in the cell cycle and the number of tumor cells that are not in the cell cycle, respectively [19]. Nevertheless, the quick growth of the tumor volume 40 days after inoculation may be explained because GF increases quickly because N_cc_ (due to the fast process of cell duplication) and/or N_n-cc_ (number of non-divisible cells enter rapidly to the cell cycle) increase, resulting in a decrease of DT. In this case, metastasis induced by DC is discarded taking into account results reported by Zhou et al. [20].

For the case of unperturbed F3II mammary carcinoma, DT is shorter because ϕ (apoptosis, necrosis, exfoliation and metastasis, mainly metastasis) and GF (quick cellular multiplication) increase, confirming that this tumor histological variety is highly aggressive and metastatic. This may be related to the finding that the high BALB/c/Cenp/mice death percentage is mainly due to the metastasis. As a result, EChT should be applied with care in this tumor histological variety.

González et al. [5] suggest that the highly aggressive and metastatic primary F3II mammary carcinoma has a major electrical conductivity and therefore its high sensitivity to DC action. This may explain, in part, the six mice deaths during and after DC application. Irreversible alterations in liver and kidney organs may be due to the metabolic burden of the products of tumor rupture, as documentd in [5]. Additionally, high amount of heat induced in the tumor by MPE should not be discarded, as predict simulations reported in [9]. Therefore, EChT should be also applied with care in this tumor histological variety.

Unlike [5], CR is not observed in any DC treated tumor in this study. Nevertheless, higher values of the overall EChT effectiveness suggest that this therapy may be addressed to highly aggressive and metastatic primary tumors, as the F3II mammary carcinoma. Despite this, it should not be categorically affirmed that EChT is safe in laboratory animals, as reported in [5].

Larger mean standard errors observed in all experimental groups may be explained from biological individuality of each mouse, physiological ulcerations in control and DC treated tumors, the diversity of response of each tumor after EChT application and the fibrosis in some DC treated tumors. These errors are marked for tumor volumes higher than 1.5 cm^3^ because their borders are very irregular. The F3II carcinoma and mouse biological individualities and the individual response of each mouse to EChT action may explain why different tumor sizes are observed at each instant of time. It is important to know that physiological ulceration is characteristic of this tumor histological variety, as reported in [10]. Furthermore, fibrosis after EChT application is observed in previous studies [5, 6].

Tumor damages induced by DC cytotoxic action during and after EChT application are well documented in the literature [1–3, 15–18, 20]. Nevertheless, the induction of necrosis (around anode and cathode) and/or apoptosis (around anode) do not necessarily lead to a complete remission of the tumor. These irreversible damages and others are necessary conditions but not sufficient to reach this tumor response type after the application of EChT or any antitumor therapy. That is why, histological analysis to verify tumor damage is not performed in this study. CR of a tumor treated with EChT or any antitumor therapy may be reached by means of longitudinal studies that combine experimental and theoretical results to know in depth how these therapies influence on TGK. That is why, this study and [5] are carried out.

The average values of $r_{a}^{2}$ close to one and the values close to zero of e_α_, e_β_, e_γ_, e_i0_, SSE, SE, PRESS, MPRESS (for m = 3), RMSE and D_max_ confirm that the MGE is feasible to describe experimental data of primary F3II mammary carcinoma treated and not treated with DC for each gender and C-I, C-II and C-III. This finding may suggest that the MGE is applicable to fit data of DC treated tumors with different values of i and t_exp_ and any geometry of electrode array (MSNEII_c_, MSNEII_nc_ and MPE).

In previous works, it has been suggested that EChT effectiveness increases for higher values of the (i/i_0_) ratio and lower value of γ [4–6]. Nevertheless, these works do not discuss how this effectiveness is affected when t_exp_ and electrode array geometry are considered. Although t_exp_ and electrode array geometry do not appear explicitly in the MGE, this study demonstrates that their influences on the F3II mammary carcinoma are implicit in the parameters i_0_ and γ, which depend also on i and the tumor histological variety growing in the host. Consequently, the (i/i_0_) ratio and parameters a_1_ and a_2_ also depend on t_exp_, electrode array geometry, tumor histological variety and type of host.

Results observed in TG2-F and TG2-M demonstrate that higher (i/i_0_) ratio, longer t_exp_ and lower value of γ correspond to the greater growth delay of primary F3II mammary carcinoma. The lowest value of γ corresponds to the longest duration of the net anti-tumor effect of the EChT induced in the tumor [4–6] and long t_exp_ is suggested in [5]. Additionally, this study indirectly confirms the hypothesis that the complete response of a solid tumor is reached from a threshold value of the (i/i_0_) ratio, named (i/i_0_)_u_. This may be argued because the complete response of the primary F3II mammary carcinoma after EChT application is not observed for the condition (i/i_0_) < (i/i_0_)_u_. González et al. [5] report experimentally that (i/i_0_)_u_ = 1.50 for this tumor histological variety. These findings demonstrate the good correspondence between the theoretical and experimental results and suggest that the primary F3II mammary carcinoma should be treated with EChT for long t_exp_ and lower i. Additionally, they confirm the existence of (i/i_0_)_u_ and corroborate the hypothesis that the more aggressive the tumor is the more sensitive to EChT. On the other hand, although tumor complete remission is not reached, significant delay of tumor growth observed in TG2-F and TG2-M may indicate that EChT constitutes a feasible option for highly aggressive and metastatic primary tumors, as in [5].

Except TG2-F and TG2-M, larger mean standard errors of the tumor volume may explain why treated groups and their respective control groups do not differ significantly when they are compared at each time instant. The latter is observed for each gender and other experimental groups. An explanation to these larger mean standard errors is given in [5]. The individual behavior of each BALB/c/Cenp mouse bearing the primary F3II mammary carcinoma observed in both control and treated groups justifies, in part, why the control group is not the true reference for treated groups, in agreement with [5, 21]. Consequently, the real control for an EChT treated tumor is the mouse itself before therapy application. That is why, the analysis of TGK should be made individually in the same mouse, bearing the same tumor type, before and after EChT application, as in [5].

Calzado et al. [9] report theoretically that all configurations of MPE induce a tumor damage percentage ≥ 80% whereas MSNEII_c_ and MSNEII_nc_ ≤ 30%. In contrast, this work shows that there are no significant differences in TGK when MPE and MSNEII_c_ are used, except for TG2-F and TG2-M (C-II and t_exp_ = 20 min). This experimental finding is observed for both genders. Consequently, two suggestions arise: MPE and MSNEII_c_ may be indistinctly used for cancer under EChT application and t_exp_ influences more on TGK delay than C-II, corroborating that larger t_exp_ and low DC doses should be recommended for highly aggressive and metastatic primary tumors.

The experimental fact that C-II does not induce high tumor damage percentage, as predicted theoretically in [9], may be explained because the F3II mammary carcinoma is a highly aggressive and metastatic primary tumor [5, 10]. It is documented that the high aggressiveness of a solid malignant tumor is due to its high intra-tumor heterogeneity and anisotropy that influence on the evolution and metastasis of the cancer, therapy resistance and the immune response [22–24]. Intra-tumor heterogeneity and anisotropy of this tumor histological variety are confirmed in [5] and in this study. Additionally, these two aspects are not included in the simulations made in [9], another reason that explains differences between these theoretical and experimental results. On the other hand, intra-tumor heterogeneity has been also linked to the stochastic spatial of cancer growth [25]. This stochastic behavior of the tumor growth is a direct consequence of the large biological individuality of DC treated and untreated tumors, as confirmed in this study and in [4–6].

Calzado et al. [9] report that higher values of the temperature and the heat generated by MPE influence more on the high tumor damage percentages than the electrochemical processes induced in the tumor by the geometries of electrode arrays. In contrast, this study suggests that these two physical quantities do not provoke significant delay on TGK in the experimental groups, in which C-II is used. We do not discard that the temperature and the heat induced in this tumor histological variety influence on unexpected EChT effectiveness for C-II, as predicted theoretically in [9]. This hypothesis may be justified because high values of these two physical quantities may induce an inflammatory process in the tumor. It has been documented that the inflammation induced in tumors is linked with their growth and metastasis [26]. Therefore, we should be very careful with the use of MPE.

Despite the most accepted antitumor mechanism of EChT is electrochemical [2, 16], acid and basic pH fronts that induce MPE overlap rapidly due to the small separation distance in each pair of electrodes. This rapid overlapping of acidic and basic pH fronts generated by MPE does not occur when the MSNEII_c_ and MSNEII_nc_ are inserted into the tumor. Recently, Calzado et al. [17] conclude that the greater separation between electrodes induce the higher area of tissue destruction. Therefore, we do not affirm completely that MPE is better than MSNEII_c_ and MSNEII_nc_ when EChT is used, in the current mode [8] and voltage mode [27].

Although the results of this study and those described in [5] agree, some differences may be mentioned. Unlike [5], in this study, it is experimentally proved the MPE effectiveness on the primary F3II mammary carcinoma. Additionally, in it is reported how MPE and MSNEII_c_ influence on MGE. This suggests that the effect induced by any geometry of electrode array on TGK may be evaluated by means of MGE. These results have no precedent in the literature. As a result, this work encourages to go deeper on how (i/i_0_)_u_ and the parameter γ depend explicitly on t_exp_, electrode array geometry [MPE, MSNEII_c_ or MSNEII_nc_], tumor histological variety, host type, number of times that EChT is repeated and combination of EChT with any antitumor therapy.

The results of this study and those reported in [5, 8, 9, 17, 18, 27] suggest that the growth delay of the primary F3II mammary carcinoma may be increased optimizing MSNEII_c_, MSNEII_nc_ and MPE geometries and/or combining EChT with other physical therapies [15, 28] and/or other antitumor therapies. For instance, a new question arises: can MGE be used to fit tumor gowth kinetics treated with combined therapies (i.e., direct current and immunotherapy/chemotherapy)?

The aforementioned may be important to plan and personalize EChT in order to reach the complete remission or the stationary partial response (cancer can be turned into a controlled chronic disease) of any tumor histological variety growing in a specific host. This cannot be obtained if we focus separately in theoretical or experimental results. That is why, we addresses our efforts to report works that integrate both results, as this study, which confirms that the knowledge and therapeutic of the cancer is in the frontier of oncology, mathematics, physics, biophysics and other sciences, as the results reported in [9, 14, 27, 29] suggest. In addition, this integrated analysis of experimental results and those that provide mathematical modeling will ensure communication in the shortest time between basic and clinical science and the proposal of an optimal personalized therapy.

## Conclusions

In conclusion, the electrochemical therapy may be potentially addressed to highly aggressive and metastic primary F3II murine mammary carcinoma and the modified Gompertz equation may be used to fit data of this direct current treated carcinoma. Additionally, electrochemical therapy effectiveness depends on the exposure time, geometry of multiple-electrodes and ratio between the direct current intensity applied and the polarization current induced in the tumor.

## Data Availability

Data used in this study cannot be shared because some of the researchers are linked to pharmaceutical and biotechnological Cuban industries by a research contract that includes sensitive experiments. Consequently, this research is under a confidentiality agreement signed for 10 years. Thus, none of the authors of this manuscript may reveal original data. Original data is kept in Bioelectricity group, Research and Innovation Department, National Center of Applied Electromagnetism, Oriente University. This confidentiality agreement is protected by the Cuban Copyright Law (National Legislature Parliament of the Cuba Republic; Law 14; December 28, 1977; chapters IIII, VII, IX, X), the Invention Law (Official Gazette of the Cuba Republic; Government Decree No. 290; February 1, 2012; pages 9–27; ISSN 1682–7511) and the Work Code includes such inventions under a labor contract (Law 116 of 2014). Besides, Cuban Code of Ethics backs this argumentation. Researchers can directly contact Dr. Luis Enrique Bergues Cabrales, head of the Bioelectricity group (Centro Nacional de Electromagnetismo Aplicado (CNEA), Universidad de Oriente, Cuba) to request access to the experimental data. His email is berguesc@yahoo.com For fielding data access queries, researchers can also contact with Dra. Clara Esther Martínez Manrique, President of the Ethics Committee of CNEA, Universidad de Oriente. Her e-mail is clarita@uo.edu.cu

## Abbreviations

EChT: Electrochemical therapy
DC: Low-level direct current
TGK: Tumor growth kinetics
MGE: Modified Gompertz equation
i: Direct current intensity
t_exp_: Time of exposure of direct current
DT: Mean tumor doubling time
T_c_: Cell cycle time
ϕ: Cell loss factors
GF: Tumor growth fraction
N_cc_: Number of tumor cells in the cell cycle
N_n-cc_: Number of tumor cells that are not in the cell cycle
MSNEII: Multiple-straight needle electrodes inserted individually in the tumor
MSNEII_c_: MSNEII inserted colineally along the major axis of the tumor
MSNEII_nc_: MSNEII inserted non-colineally anywhere of the tumor
MPE: Multiple-pairs of electrodes
NaCl: Sodium chloride
CENPALAB: Centro Nacional para la Producción de Animales de Laboratorio
LABIOFAM: Laboratorios Biológicos Farmacéuticos
C-I: MSNEII_c_ formed by four collinear electrodes with alternating polarities inserted perpendicularly along the largest diameter of the tumor
C-II: MPE formed by electrode pairs arranged to 45° (electrode pair 1–2), 135° (electrode pair 3–4), 225° (electrode pair 5–6) and 325° (electrode pair 7–8)
C-III: MPE formed by electrode pairs arranged to 0° (electrode pair 1–2), 90° (electrode pair 3–4), 180° (electrode pair 5–6) and 270° (electrode pair 7–8)
CG1: Control group in which C-I is used but DC is not applied
CG2: Control group in which C-II is used but DC is not applied
TG1: Group treated with 2 mA for 10 min and C-I
TG2: Group treated with 6 mA for 20 min and C-II
TG3: Group treated with 2 mA for 10 min and C-III
TG4: Group treated with 2 mA for 10 min and C-II
TG5: Group treated with 6 mA for 10 min and C-II
TG6: Group treated with 10 mA for 10 min and C-II; CG1-F, CG2-F, TG1-F, TG2-F, TG3-F, TG4-F, TG5-F and TG6-F are these groups for females whereas CG1-M, CG2-M, TG1-M, TG2-M, TG3-M, TG4-M, TG5-M and TG6-M for the males
TGK_1_: First part comprises from the moment of inoculation of the tumor cells in the host until the day that DC is applied
TGK_2_: Second part includes from the moment of DC application until the day of sacrifice of the mice for ethical reasons
V^*^(t): Tumor volume at time t after DC treatment
α: Intrinsic growth rate of the tumor
β: Growth deceleration factor related to the endogenous antiangiogenesis processes
α^*^: modified tumor growth rate due to DC action
i_o_: Polarization current
γ: First-order exponential decay rate of the net effect induced in the solid tumor after the DC is removed
a_1_ and a_2_: Dimensionless parameters that depend on the (i/i_o_) ratio
D_max_: Maximum distance
RMSE: Root Means Square Error
SSE: Sum of squares of errors
SE: Standard error of the estimate
*r*^2^: Goodness-of-fit
$r_{a}^{2}$: Adjusted goodness-of-fit coefficient of multiple determination
PRESS: Predicted residual error sum of squares
MPRESS: Multiple predicted residual sum error of squares
$V_{j}^{*}$: *j*-th measured tumor volume at discrete time t_j_
$\widehat{V}_{j}^{*}$: *j*-th estimated tumor volume by MGE
n_1_: Number of experimental points
k: Number of parameters
$\left( {V_{j}^{*} } \right)^{\prime }$: Estimated value of $V_{j}^{*}$ when MGE is obtained without the *j*-th observation
e_α_: Estimation error of the parameter α
e_β_: Estimation error of the parameter β
e_γ_: Estimation error of the parameter γ
e_i0_: Estimation error of the parameter i_0_
N: Total number of determinations
min: Minutes
